# Myeloid-Derived Suppressor Cells in Inflammatory Arthritis

**DOI:** 10.3390/ijms27125365

**Published:** 2026-06-14

**Authors:** Daniel R. McDougle, James J. Moon, David A. Fox

**Affiliations:** 1 Division of Rheumatology, Department of Internal Medicine, University of Michigan, Ann Arbor, MI 48109, USA; mcdougda@med.umich.edu; 2 Department of Pharmaceutical Sciences, University of Michigan, Ann Arbor, MI 48109, USA; 3 Biointerfaces Institute, University of Michigan, Ann Arbor, MI 48109, USA; 4 Department of Biomedical Engineering, University of Michigan, Ann Arbor, MI 48109, USA; 5 Department of Chemical Engineering, University of Michigan, Ann Arbor, MI 48109, USA

**Keywords:** myeloid-derived suppressor cells, rheumatoid arthritis, inflammatory arthritis, immune regulation, cytokine signaling, myeloid plasticity

## Abstract

Myeloid-derived suppressor cells (MDSCs) are a heterogeneous group of immature myeloid cell populations with potent immunosuppressive activity. MDSCs accumulate during states of chronic inflammation in response to inflammatory cytokine signaling that triggers emergency myelopoiesis in the bone marrow. In rheumatoid arthritis and experimental models of inflammatory arthritis, MDSCs were initially thought to serve as a regulatory checkpoint that limits excessive inflammation. However, subsequent studies have shown that these cells can either alleviate or worsen arthritis depending on immunophenotype, disease timing, microenvironment, cytokines/chemokines, and transcriptional states. Taken together, the seemingly paradoxical roles of MDSCs in inflammatory arthritis likely reflect a highly plastic and context-dependent myeloid continuum. This review examines current knowledge of MDSCs in inflammatory arthritis, highlighting the conditions that direct their functional diversity and the factors that determine whether they alleviate or exacerbate disease. We also discuss emerging therapeutic strategies and emerging concepts to better understand these immune cell populations in the context of inflammatory arthritis.

## 1. Introduction

Myeloid-derived suppressor cells (MDSCs) are a heterogeneous population of pathologically activated immature myeloid cells that accumulate under conditions of chronic inflammation where they mediate potent immunosuppressive functions [1,2]. Under normal physiological conditions, bone marrow-derived immature myeloid cells differentiate into granulocytes, macrophages, and dendritic cells, with minimal production of MDSCs. However, in pathological settings such as cancer, infection, and chronic systemic autoimmune disease, this differentiation process becomes dysregulated, promoting the expansion of MDSCs [1,2].

Functionally, MDSCs are distinct from their differentiated myeloid counterparts, exhibiting unique genomic, biochemical, and metabolic programs that confer potent immunosuppressive capacity [2,3,4]. MDSCs suppress both innate and adaptive immunity through complex, interdependent mechanisms, including metabolic/enzymatic pathways (e.g., arginase-1, iNOS), cytokine and chemokine signaling (e.g., IL-10, TGF-β), and cell–cell interactions (e.g., PD-L1) [3,5]. Moreover, the immunomodulatory effects of MDSCs are nuanced and context-dependent, varying across tissues and microenvironments.

The primary focus of this review is to provide an overview of the fundamental aspects of MDSCs’ biology and the mechanisms that govern their immunoregulatory functions in inflammatory arthritis. Given the relatively limited literature specific to MDSCs in the setting of inflammatory arthritis, we have also included relevant parallel reports detailing mechanistic pathophysiology of other autoimmune diseases, including systemic lupus erythematosus (SLE), Sjogren’s syndrome (SS) and multiple sclerosis. To maintain autoimmune disease-specific relevance, studies pertaining to cancer, infection, and transplantation were generally excluded. We specifically address the conflicting evidence regarding their roles in inflammatory arthritis, where MDSCs have been implicated in both disease amelioration and progression. Additionally, we examine current and emerging therapeutic strategies targeting these populations and highlight key unanswered questions in the field.

## 2. Literature Search Strategy

A literature search was performed using PubMed to identify studies investigating MDSCs in RA and experimental arthritis models. Briefly, Medical Subject Headings (MeSH) and keyword-based searches of titles and abstracts were performed using the following terms: (“Myeloid-Derived Suppressor Cells”[Mesh] OR “myeloid-derived suppressor cell*”[tiab] OR MDSC*[tiab] OR “myeloid suppressor cell*”[tiab] OR “immature myeloid cell*”[tiab] OR “CD11b+Gr-1+”[tiab] OR “Gr-1+CD11b+”[tiab]) AND (“Arthritis, Rheumatoid”[Mesh] OR “rheumatoid arthritis”[tiab] OR “collagen-induced arthritis”[tiab] OR CIA[tiab] OR “antigen-induced arthritis”[tiab] OR AIA[tiab] OR “collagen antibody-induced arthritis”[tiab] OR CAIA[tiab] OR SKG[tiab] OR “K/BxN”[tiab] OR “experimental arthritis”[tiab]). Studies involving human RA samples as well as murine experimental arthritis models, including CIA, AIA, CAIA, SKG, and K/BxN systems, were included. Reference lists from relevant original articles and review papers were additionally screened to identify further studies not captured in the primary database search. Studies involving human RA samples as well as murine experimental arthritis models, including CIA, AIA, CAIA, SKG, and K/BxN systems, were included. Papers were excluded if they failed to verify T-cell-suppressive function, lacked mechanistic evaluation of MDSC-mediated effects, or only briefly mentioned MDSCs in associative or descriptive contexts. Reference lists from relevant original articles and review papers were additionally screened to identify additional studies not captured in the primary database search.

## 3. Historical Overview and Subset Diversity of MDSCs

### 3.1. From Cancer Immunology to Autoimmunity

Initial reports of suppressive splenic immune cells originated in cancer immunology, where these populations were demonstrated to promote tumor survival via suppression of cell-mediated immunity [6]. Subsequent, mechanistic studies demonstrated the presence of arginase-1 (Arg-1)-producing myeloid cells negatively regulated T-cell responses, thereby promoting tumor growth [7]. Formal nomenclature of myeloid-derived suppressor cells (MDSCs) was adopted in 2007 to describe the myeloid lineage origin and capacity to promote tumor-associated T-cell tolerance [1]. Subsequent studies have demonstrated that these immature myeloid cells accumulate in blood, lymph nodes, bone marrow, and tumor sites, where they profoundly suppress T-cell- and natural killer (NK) cell-mediated antitumor immunity [8]. MDSC pathobiology has evolved with the recognition that chronic systemic inflammatory conditions promote the accumulation of MDSCs which down-regulate immune surveillance, and with the establishment of MDSCs as multifaceted promoters of tumor growth, angiogenesis and metastasis [8,9]. 

Interest in MDSCs in autoimmune and autoinflammatory diseases emerged from the hypothesis that chronic inflammatory states would promote emergency myelopoiesis driving the expansion of MDSCs. Indeed, the first evidence supporting a role for MDSCs in autoimmune disease emerged from studies in Experimental Autoimmune Encephalomyelitis (EAE), where MDSCs were shown to expand during disease and were powerful suppressors of activated T cells [10]. Subsequent studies demonstrated that MDSCs mediate immunoregulatory roles in a wide range of autoimmune diseases, including inflammatory bowel disease [11], type 1 diabetes [12], autoimmune uveitis [13], collagen-induced arthritis (CIA), and systemic lupus erythematosus [14]. However, it has become increasingly clear that functions of MDSCs are highly context-dependent. Rather than acting solely as immunosuppressive regulators, MDSCs can either attenuate or promote inflammatory diseases depending on their activation state, subset composition, and the local tissue microenvironment [15].

Early reports on the role of MDSCs in RA and experimental inflammatory arthritis revealed paradoxical findings, with evidence supporting both amelioration [16,17] and exacerbation [18,19] of disease pathogenesis. Notably, these diametrically opposed effects are not unique to inflammatory arthritis but represent a conserved feature in early reports of murine EAE [20,21], SLE [14,22], and SS [23,24], which continue to be active areas of research. Elucidating the mechanisms governing MDSC heterogeneity and functional plasticity is therefore essential for understanding their role in autoimmune disease pathogenesis and for guiding therapeutic targeting. The phenotypic characteristics and mechanistic pathways underlying these divergent functions are discussed below.

### 3.2. Development and Immunophenotype of MDSCs

**Figure 1:**
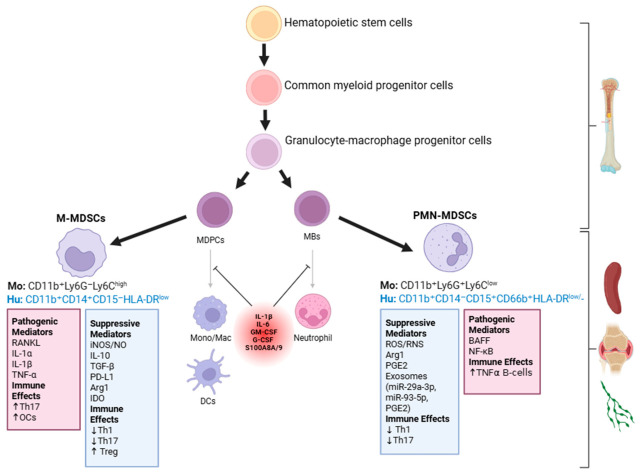
Developmental origin and functional roles of myeloid-derived suppressor cell (MDSC) subsets in inflammatory arthritis. Hematopoietic stem cells in the bone marrow differentiate into common myeloid progenitors and subsequently granulocyte–macrophage progenitors, which give rise to two progenitor branches: MDPCs and MBs. Under inflammatory conditions (IL-6, GM-CSF, and S100A8/A9), progenitors expand and differentiate into M-MDSCs and PMN-MDSCs and take residence in spleen, lymph nodes and inflamed joints, where they exert both protective and pathogenic roles in inflammatory arthritis. Black arrows indicate hematopoietic lineage progression and differentiation pathways leading to M-MDSC and PMN-MDSC development, whereas gray arrows indicate proposed cellular interactions and cytokine-mediated influences within the inflammatory microenvironment. Schematic drawn with Biorender.com. Abbreviations: MDPCs, monocyte–dendritic progenitor cells; MBs, myeloblasts; Mono/Mac, monocytes/macrophages; DCs, dendritic cells; PMNs, polymorphonuclear neutrophils; PMN-MDSCs, polymorphonuclear myeloid-derived suppressor cells; M-MDSCs, monocytic myeloid-derived suppressor cells; IL-1α, interleukin-1 alpha; IL-1β, interleukin-1 beta; IL-6, interleukin-6; TNF-α, tumor necrosis factor alpha; GM-CSF, granulocyte–macrophage colony-stimulating factor; G-CSF, granulocyte colony-stimulating factor; BAFF, B-cell-activating factor; RANKL, receptor activator of nuclear factor κB ligand; NF-κB, nuclear factor kappa B; iNOS, inducible nitric oxide synthase; NO, nitric oxide; ROS, reactive oxygen species; RNS, reactive nitrogen species; Arg1, arginase-1; IDO, indoleamine 2,3-dioxygenase; PD-L1, programmed death-ligand 1; PGE2, prostaglandin E2; TGF-β, transforming growth factor beta; Th1, T helper 1 cells; Th17, T helper 17 cells; Treg, regulatory T cells; OCs, osteoclasts; S100A8/A9, S100 calcium-binding proteins A8 and A9; miR, microRNA; Mo, mouse; Hu, human.

MDSCs are classified based on origin, immunophenotype and experimentally validated demonstration of suppressive function (Figure 1). Importantly, phenotypic identification alone is insufficient to define MDSCs, as numerous immunophenotypic and biochemical markers overlap with those of neutrophils and monocytes [25]. Thus, T-cell suppression assays are necessary to confirm MDSC-suppressive functions and distinguish these cells from their differentiated counterparts [26].

Under steady-state conditions, bone marrow-derived immature myeloid cells undergo terminal differentiation into granulocytes, macrophages, and dendritic cells. In contrast, chronic inflammatory conditions characterized by elevated levels of granulocyte–macrophage colony-stimulating factor (GM-CSF), granulocyte colony-stimulating factor (G-CSF), interleukin-1 beta (IL-1β), interleukin-6 (IL-6), and S100 calcium-binding protein A8/A9 (S100A8/A9) alarmins drive emergency myelopoiesis, leading to the accumulation of immature myeloid cells (Figure 1) [27,28,29]. These inflammatory signals activate intracellular pathways, including janus kinase/signal transducer and activator of transcription 3 (JAK/STAT3), nuclear factor kappa B (NF-κB), and phosphoinositide 3-kinase/protein kinase B (PI3K/AKT), which promote cell survival, metabolic reprogramming, and the acquisition of immunosuppressive effector functions [30].

MDSCs are grouped into two major sub-populations that include monocytic MDSCs (M-MDSCs), which resemble inflammatory monocytes, and polymorphonuclear MDSCs (PMN-MDSCs) akin to immature neutrophils [31,32]. In mice, subsets are distinguished by differential expression of Ly6C and Ly6G markers with the M-MDSC immunophenotype designated as CD11b^+^Ly6C^hi^Ly6G^−^, while PMN-MDSCs are CD11b+Ly6C^low^Ly6G^+^ [32]. In humans, M-MDSCs are typically defined as CD11b^+^CD33^+^CD14^+^HLA-DR^low/−^ CD15^−^, whereas PMN-MDSCs are CD11b^+^CD33^+^CD15^+^CD66b^+^HLA-DR^−^CD14^−^ [33]. A third subset has been recognized in humans, termed early-stage MDSCs (eMDSCs), which is characterized by the absence of lineage-specific markers and low/absent HLA-DR expression (Lin^−^ HLA-DR^−^ CD33^+^), reflecting an immature myeloid phenotype [30]. However, eMDSCs have yet to be functionally characterized in rodents.

In cancer biology, single-cell RNA sequencing (scRNA-seq) has emerged as a powerful tool for resolving MDSC heterogeneity. These efforts have revealed an MDSC-specific gene signature and CD84 and LOX-1 expression distinguishing immunosuppressive MDSCs from classical neutrophils [34,35]. Studies in humans and tumor-bearing mice distinguished an activated, immunosuppressive PMN-MDSC population within the tumor microenvironment from an inactive circulating counterpart [36]. Notably, M-MDSC scRNA-seq revealed five transcriptionally distinct M-MDSC subsets that showed different levels of immunosuppressive activity depending on the stage of infection in sepsis [37].

In the context of autoimmune-mediated diseases, high-dimensional profiling of MDSCs remains in its early stages. In experimental arthritis, MDSC transcriptomics have been evaluated using bulk microarray analysis of sorted CD11b^+^Gr-1^+^ cells from arthritic SKG mice, revealing tissue-specific transcriptional programs distinguishing immunosuppressive and osteoclastogenic joint-resident MDSCs [38]. In human RA, large-scale single-cell atlases of synovial tissue identified IL-1β^+^ pro-inflammatory monocyte populations expanded in inflamed joints that share transcriptional features with M-MDSCs, although these populations were not validated or annotated as MDSCs [39,40]. To date, there are no reports of MDSC subset profiling in human RA or experimental arthritis using high-dimensional single-cell profiling approaches.

### 3.3. Subset-Associated Functional Divergence

PMN-MDSCs are frequently reported to exhibit robust immunosuppressive activity in vitro and in adoptive transfer models of inflammatory arthritis. In CIA, PMN-MDSC transfer reduced joint inflammation and dampened both T helper 1 (Th1) and T helper 17 (Th17) responses while decreasing pro-inflammatory cytokines [41]. Notably, M-MDSCs showed no therapeutic effect in the same study, highlighting subset-specific protective capacity [41]. PMN-MDSCs generated through S100A8/A9 bone marrow stimulation were potent suppressors of Th17 and were also found to promote regulatory T-cell (Treg) expansion [42]. In the proteoglycan-induced arthritis (PGIA) model, bone marrow-derived PMN-MDSCs ameliorated arthritis through nitric oxide (NO)-dependent suppression of T-cell proliferation and reduction in antigen-specific antibody responses [17].

M-MDSCs have been shown to exhibit greater functional plasticity with context-dependent outcomes. Under protective conditions, M-MDSCs are suppressive through NO production, PGE2 secretion, and cell-contact mechanisms that inhibit B-cell proliferation and autoantibody production, reducing joint inflammation and rescuing CCR2-deficient mice [43]. Paradoxically, M-MDSCs have also been shown to promote Th17 differentiation and facilitate disease progression via IL-1β and TNF-α pathways [44]. Selective depletion of M-MDSCs ameliorated disease, while adoptive transfer facilitated progression [44]. Collectively, these findings highlight the context-dependent and potentially divergent roles of MDSC subsets in inflammatory arthritis.

## 4. Context-Dependent Immunoregulatory Functions of MDSCs in Experimental Arthritis

### 4.1. MDSC-Mediated Suppression of T-Cell Responses

#### 4.1.1. Protective Mediators

On the basis of the existing literature pertaining to experimental inflammatory arthritis, NO and interleukin 10 (IL-10) have emerged as two essential mediators for the suppressive functions of MDSCs in inflammatory arthritis. Specifically, IL-10 has been shown to promote the expansion of forkhead box P3-expressing (Foxp3^+^) Tregs while simultaneously inhibiting pathogenic Th1 and Th17 responses [45]. Additional studies showed that adoptive transfer of MDSCs derived from IL-10-deficient mice failed to attenuate CIA [45]. In parallel studies, pharmacological inhibition of inducible nitric oxide synthase (iNOS) using L-NMMA exacerbated arthritis severity in vivo, demonstrating that endogenous MDSCs rely on NO to restrain immune-mediated joint damage [42]. Together, these findings highlight IL-10-driven immune modulation and NO-mediated effector suppression as complementary and essential pathways through which MDSCs limit pathogenic T-cell responses and maintain immune homeostasis in inflammatory arthritis. In some contexts, high expression of Arginase-1 (Arg-1) has been shown to promote anti-inflammatory effects [45], although this enzyme has also been implicated in pro-arthritic functions as discussed below.

Parallel experimental autoimmune murine models (AEA, SLE, SS) have similarly been shown to conserve T-cell-targeted protective mechanisms. In EAE, PGE2-differentiated PMN-MDSCs reduced Th17 and IFN-γ NK cell infiltration, whilst expanding Tregs [46]. In SLE, PD-L1-expressing MDSCs expanded Tregs and regulatory B cells, reducing autoantibody levels and renal pathology [47]. In experimental SS, AhR activation was shown to potentiate PMN-MDSC-suppressive function on CD4^+^ T cells and attenuate salivary gland pathology [48]. These parallels suggest that MDSCs share a suppressive phenotype across diverse autoimmune diseases.

#### 4.1.2. Protective Transcription Factors

Early Growth Response transcription factors 2 and 3 (EGR2 and EGR3) are enriched in joint-resident MDSCs and are associated with a suppressive gene program, in part via induction of suppressor of cytokine signaling 1 (SOCS1) which inhibits signal transducer and activator of transcription 1 (STAT1) and STAT3 pathways [38]. Although not expressed by MDSCs, Foxp3 represents a critical downstream transcription factor of MDSC-mediated immunoregulation, as MDSCs promote the expansion of Foxp3^+^ Tregs via IL-10-dependent mechanisms. Collectively, these findings position transcriptional control as a key determinant of MDSC-suppressive capacity, integrating intrinsic regulatory programs with extrinsic signals to maintain immune homeostasis and limit pathogenic inflammation [45].

### 4.2. MDSC-Mediated T-Cell Pathogenesis in Inflammatory Arthritis

#### 4.2.1. Pathogenic Mediators

In experimental inflammatory arthritis, the Arg-1-dependent pathway plays a central role in promoting pathogenic immune responses. Several studies have demonstrated that MDSCs are a potent source of IL-1β that promotes Th17 differentiation [19,44]. Consistent with this, blockade of IL-1 signaling using an IL-1 receptor antagonist (IL-1Ra) significantly reduced MDSC-mediated IL-17A production [19]. IL-6 has emerged as a key cytokine that programs MDSCs toward a pro-inflammatory phenotype through induction of Arg-1. In contrast to its classical immunosuppressive role in cancer, Arg-1 can promote Th17 polarization via polyamine metabolism and subsequent activation of the transforming growth factor beta (TGF-β) and SMAD family member 3 (SMAD3) signaling in T cells [49]. In parallel, TNF-α serves as a major driver of both MDSC accumulation and pathogenic polarization, promoting MDSC proliferation, skewing cells toward an inflammatory phenotype, and enhancing B-cell activation through induction of B-cell-activating factor (BAFF) [50].

In experimental murine models of SLE, the Arg-1/Th17 pathogenic axis is consistently conserved. Early reports have shown that MDSC-derived Arg-1 promotes Th17 differentiation in an Arg-1-dependent manner that promotes renal injury in a humanized model [14]. Subsequent studies showed that MDSC-derived Arg-1 upregulates miR-322-5p, which activates TGF-β signaling to shift the Th17/Treg ratio toward Th17 predominance [51]. In EAE, MDSCs similarly promote Th17 via IL-1β in a mechanism nearly identical to that described in CIA [21]. Subsequent studies demonstrated that EAE induction mobilizes granulocytic MDSCs to the lungs where they promote Th17 polarization critical for EAE induction [52]. In experimental SS, it was demonstrated that adoptive transfer of MDSCs exacerbated SS-like disease in NOD mice by suppressing Th2 responses, shifting the T-cell balance toward pathogenic subsets [23]. Subsequent studies showed that FcγRIIIA-mediated glycolytic reprogramming of MDSCs upregulated the Th17/Treg ratio and correlated with disease activity, which was reversible by glycolysis inhibition with 2-deoxy-D-glucose [53]. The conservation of this pathogenic axis across autoimmune diseases suggests it represents a fundamental feature of MDSC biology in autoimmunity rather than a disease-specific phenomenon.

#### 4.2.2. Pro-Inflammatory Transcriptional Programs

Pathogenic transcriptional programs in MDSCs promote inflammatory arthritis through the induction of Th17 polarization and osteoclastogenesis [54]. Activation of STAT3 has been demonstrated to be a key mediator of MDSC-driven Th17 responses in inflammatory arthritis [49]. STAT3 activation reprograms MDSCs from potentially regulatory cells into pathogenic effectors of inflammation, in part through induction of Arg-1 [49]. MDSCs further sustain Th17-mediated inflammation by enhancing expression of retinoic acid receptor-related orphan receptor gamma t (RORγt) via IL-1β-dependent mechanisms [19]. In parallel, osteoclastogenic potential is driven by activation of the non-canonical NF-κB pathway (Nfkb2 and Relb) which was demonstrated to be essential for the differentiation of MDSCs into bone-resorbing osteoclasts and subsequent joint destruction [38].

Aberrant STAT3 activation appears conserved across other autoimmune diseases. In EAE, genetic disruption of Stat3 in myeloid lineage cells abrogated disease, through disruption of myeloid antigen presentation which is essential for encephalitogenic T-cell differentiation [55]. Non-canonical STAT3 activity further sustained pathogenic Th17 proliferation and cytokine responses [56], while E3 ubiquitin ligase Hectd3-deficient mice showed reduced STAT3 polyubiquination to drive pathogenic Th17 generation [57]. In experimental murine SLE, STAT3/Arg-1 signaling in MDSCs promoted Th17 differentiation in an Arg-1-dependent manner [14], with aberrant p-STAT3 hyperactivation driven by miRNA-mediated suppression of STAT3 inhibitors SOCS1, PTEN, and PIAS3 [58].

### 4.3. Microenvironmental and Contextual Determinants of MDSC Function

#### 4.3.1. Microenvironmental Cues

In inflammatory arthritis, the alarmin S100A8/A9 serves as a key regulator of MDSC-mediated T-cell polarization in inflammatory arthritis by modulating the Treg/Th17 balance in a context-dependent manner. Short-term exposure to S100A8/A9 promotes classical pro-inflammatory activation of myeloid cells, whereas sustained exposure in the CIA model was shown to reprogram myeloid precursors into immunosuppressive MDSCs [42]. This effect was demonstrated to be mediated primarily through TLR4 signaling, particularly within joint-draining lymph nodes where local concentrations of S100 proteins are elevated. Chronic elevation of S100A8/A9 promoted MDSC-mediated expansion of Foxp3^+^ Tregs while suppressing Th17 differentiation and effector cytokine production, including IL-17 and interleukin 22 (IL-22) [42]. Interestingly, deficiency of S100A8/A9 impaired Treg expansion and exacerbated arthritis severity [42]. Together, these findings suggest chronic S100A8/A9 → TLR4 activation acts as a critical microenvironmental signal that integrates inflammatory duration with regulatory MDSC function.

In other autoimmune diseases, the microenvironmental cues governing MDSC functional polarization are diverse, likely reflecting the specific pathology of each disease. In EAE, the CNS milieu itself reprograms infiltrating PMN-MDSCs into functional suppressors via STAT3-dependent signaling, with local IFN-γ from activated T cells serving as an essential signal [59]. In SLE, the inflammatory milieu paradoxically eliminates regulatory G-MDSCs through ROS-dependent extracellular trap formation [60], while TLR7-activated Notch1 signaling in the bone marrow drives aberrant MDSC expansion [61] and MDSC-derived S100A8/A9 feeds forward through TLR7 to amplify myeloid cell activation [60]. Collectively, these findings suggest that the microenvironmental control over MDSC function is dictated by the unique tissue and immunological context of each disease.

#### 4.3.2. Influence of Disease Stage and Temporal Dynamics

As shown in Table 1, in experimental inflammatory arthritis, early intervention with MDSC transfer (days 0–21) consistently reduces inflammation, decreases Th17 responses, and improves clinical outcomes [17,41,43,62,63]. In contrast, during established disease, MDSCs are prone to develop pro-arthritic phenotypes (Table 2). For example, MDSCs isolated from late-stage CIA (day 35) restored disease following anti-Gr-1 depletion through IL-1β-mediated Th17 promotion [19]. This temporal shift suggests that chronic inflammatory exposure has the potential to reprogram MDSCs toward pathogenic phenotypes and provides potential explanation why endogenous MDSC expansion fails to control disease progression despite accumulation at peak inflammation.

#### 4.3.3. Tissue Microenvironment and Osteoclastogenic Potential

In the context of inflammatory arthritis, the inflammatory microenvironment provides an additional regulatory layer that can promote MDSC transformation into osteoclasts. Early reports showed that intratibial transfer of MDSCs from CIA mice resulted in osteoclastogenesis via a reversible NF-κB and IL-1 α axis pathway, which was significantly reduced in MDSCs derived from naïve mice [18]. In subsequent studies, differential gene analyses of bulk MDSCs from the bone marrow, spleen and joint were performed using microarray analysis, which showed that joint MDSCs expressed genes of the NF-κB non-canonical pathway (Nfkb2 and Relb) and promoted osteoclast differentiation and destructive arthritis with intra-articular injection [38]. Another MDSC-dependent osteoclastogenic pathway was shown to be mediated by the glycoprotein dickkopf-1 (DKK-1), a Wnt pathway antagonist involved in bone remodeling, which promoted MDSC expansion and differentiation into osteoclasts via suppression of the Wnt/β-catenin pathway [64].

Beyond intrinsic transcriptional reprogramming, extrinsic cellular interactions within the joint microenvironment also regulate MDSC osteoclastogenic potential. B cells from RA patients augment the osteoclastogenic capacity of MDSCs, with switched memory B cells exerting the most pronounced effect followed by CD21-low and naïve B cells, while MDSCs from B-cell-deficient mice exhibited diminished osteoclast differentiation capacity [65]. Complementarily, M-MDSCs program Th17 cells toward a pro-osteoclastogenic phenotype via RANKL-RANK signaling and selective depletion of M-MDSC-ameliorated osteoclastogenesis [66].

Interestingly, extrinsic environmental factors may also influence MDSC biology as high-humidity housing exacerbated CIA in association with splenic MDSC expansion and elevated oxidative stress although the mechanistic link remains correlative [67].

### 4.4. MDSC Interactions with Other Immune Cells

#### 4.4.1. MDSC Regulation of B Cells

Beyond T-cell suppression, MDSCs have also been shown to regulate other aspects fo humoral immunity in inflammatory arthritis. M-MDSCs suppress B-cell proliferation and autoantibody production through NO, PGE2, and cell-contact mechanisms [43]. Conversely, PMN-MDSCs can promote pathogenic B-cell responses through BAFF secretion, activating TNF-α-producing B cells via Bruton’s tyrosine kinase (BTK) and NF-κB signaling, thereby exacerbating disease [50]. This dual capacity to either suppress or activate B-cell responses further illustrates MDSCs’ functional plasticity.

In SLE, MDSC–B-cell interactions are similarly paradoxical. PMN-MDSCs promoted IFN-I signaling activation of B cells through the lncRNA NEAT1-BAFF axis, with NEAT1 deficiency significantly attenuating lupus symptoms [68]. Conversely, adoptive transfer of MDSCs in the Sanroque lupus model expanded regulatory B cells via iNOS, reducing anti-dsDNA antibodies and proteinuria [22]. This dual capacity to either activate or suppress B-cell responses through distinct molecular pathways mirrors the findings in CIA and further underscores the context-dependent nature of MDSC–B-cell crosstalk.

#### 4.4.2. Paracrine Regulation via MDSC-Derived Exosomes

Emerging evidence highlights MDSC-derived exosomes as an additional regulatory mechanism extending beyond the other more well-characterized immunomodulatory functions. PMN-MDSC exosomes transferred immunomodulatory cargo including miR-29a-3p (targeting T-bet to suppress Th1) and miR-93-5p (targeting STAT3 to suppress Th17), resulting in reduced arthritis and joint destruction [69]. Exosomal PGE2 promoted IL-10-producing regulatory B cells while reducing pathogenic plasma cells and T follicular helper cells, leading to decreased serum IgG and attenuated disease [70]. These findings suggest that MDSC paracrine regulation may offer therapeutic opportunities independent of cellular transfer.

#### 4.4.3. Specialized MDSC-like Populations

An osteoclast precursor-like MDSC population with phenotype of CD11b^−/lo^ Ly6C^hi^ was previously reported. This unique population demonstrated potent suppressive properties, requiring T-cell-derived IFN-γ for NO-mediated activity, that ameliorated inflammatory arthritis without increased erosive disease [71]. These results demonstrated protective functions, distinct from conventional MDSC subsets and highlighting the heterogeneity within suppressive myeloid populations. As of the time of this review, there are no direct CD11b^−/lo^ Ly6C^hi^ counterparts in humans, in part due to lack of Ly6C and requisite CD11b positivity for human MDSC phenotyping. Nonetheless, immature myeloid populations, such as eMDSCs (Lin^−^ HLA-DR^−^ CD33^+^) and monocytic MDSCs with osteoclastogenic potential, may represent functional counterparts.

**Table 1:** Summary of studies demonstrating protective functions of MDSCs in inflammatory arthritis.

Model	Strain; Species	MDSC Source	Isolation Method	MDSC Subset(s)	Treatment and Timing	Key Mechanism	Outcome	Ref
CIA	DBA/1 mice	Spleen (CIA mice)	MACS	Bulk MDSCs (CD11b^+^Gr-1^+^)	IV ACT (2 × 10^6^) Day 25. Early-arthritis.	↓ CD4^+^ T cells, ↓ Th17, ↑ IL-10	Reduced joint inflammation	[16]
CIA	DBA/1 mice	Spleen (CIA mice)	FACS	PMN-MDSCs (CD11b^+^Ly6C^−^Ly6G^+^); M-MDSCs (CD11b^+^Ly6C^+^Ly6G^−^)	IV ACT (2 × 10^6^) Day 14. Pre-arthritis.	↓ Th1/Th17, ↓ serum cytokines	PMN-MDSCs effective; M-MDSCs not therapeutic	[41]
SKG arthritis	SKG mice	BM (arthritic SKG mice)	MACS	Bulk MDSCs (CD11b^+^Gr-1^+^)	IV ACT (2 × 10^6^) Days 7, 17, 27. Pre-arthritis.	Tofacitinib (JAK1/3 inhibition) ↑ MDSCs	Reduced arthritis severity	[63]
CIA	DBA/1 mice	Spleen (CIA mice)	MACS	M-MDSCs (CD11b^+^Ly6C^+^Ly6G^−^)	IV ACT (2.5 × 10^5^); IP ACT (1.5 × 10^6^) Day 14, then q5d. Pre-arthritis.	NO, PGE2, cell contact; ↓ B cells	Reduced inflammation; rescued CCR2^−^/^−^ mice	[43]
CIA	DBA/1 mice	Spleen (CIA mice)	FACS	Bulk MDSCs (CD11c^−^CD11b^+^Gr-1^+^)	IV ACT (5 × 10^5^) Day 21. Early-arthritis.	IL-10 → Treg induction; ↓ Th17/Th1	Attenuated CIA; IL-10 KO ineffective	[45]
CIA	DBA-1 mice	Spleen (3AC treated mice)	FACS	Bulk MDSCs (CD11b+Gr-1)	IV ACT (8 × 10^5^) Day -1; Day 20. Pre-arthritis.	SHIP1 inhibition → PI3K/AKT → MDSC expansion; 3AC-conditioned MDSCs suppress disease	Reduced CIA incidence and severity; naïve MDSCs ineffective	[72]
PGIA	BALB/c mice	BM (cultured; naive)	MACS	BM-MDSCs (CD11b^+^Ly6G^+^Ly6C^low^)	IP (1 × 10^7^) Day 15. Early-arthritis.	NO, ↓ T-cell proliferation	Reduced arthritis and antibodies	[17]
CIA	C57BL/6 mice	BM (naive)	MACS	BM-MDSCs (CD11b+Gr-1)	IV ACT (2 × 10^6^) Days 21, 28. Early-arthritis.	S100A8/A9 MDSCs; ↓ Th17, ↑ Treg	Suppressed T-cell responses	[42]
CIA	DBA/1 mice	BM (CIA mice)	Exosome purification	PMN-MDSC exosomes	IV 100 µg exosomes Days 18, 24. Pre-arthritis.	miR-29a-3p, miR-93-5p; ↓ Th1/Th17	Reduced arthritis and joint damage	[69]
CIA	DBA/1 mice	Spleen	Exosome purification	PMN-MDSC exosomes	IV 100 µg exosomes Days 18, 24. Pre-arthritis.	PGE2 → ↑ Bregs; ↓ plasma cells	Reduced arthritis, ↓ anti-CII IgG	[70]
SKG arthritis	SKG/Rag2^−^/^−^	BM	MACS; FACS	OCP-MDSCs CD11b^−/lo^Ly6C^hi^	IV ACT (4 × 10^5^) Day 0. Pre-arthritis.	NO; IFN-γ dependent	Ameliorates arthritis without erosion	[71]
RA	Human	Synovial fluid	Arthrocentesis	PMN-MDSCs > M-MDSCs	Co-culture with T cells. N/A.	Likely NO-mediated	Suppresses T-cell responses	[73]
PGIA	BALB/c mice	Spleen + synovial fluid	MACS	CD11b^+^Gr-1^+^	DC/T-cell co-culture.	↑ iNOS, ↑ ROS	Suppresses DC maturation	[74]
GPI arthritis	DBA/1 mice	LN (GPI mice)	FACS	CD369^+^CD11b^+^Gr-1^+^ MDSCs	FTY720 + GPI antigen × 5 days. Pre-arthritis.	CD369^+^ MDSCs with high T-cell-suppressive capacity	Expanded suppressive MDSCs; immune tolerance	[75]

Symbols: ↑ increased; ↓ decreased; → promoted, induced, or resulted in.

**Table 2:** Summary of studies demonstrating pathogenic functions of MDSCs in inflammatory arthritis.

Model	Strain; Species	MDSC Source	Isolation Method	MDSC Subset(s)	Treatment and Disease Timing	Key Mechanism	Outcome	Ref
CIA	DBA/1 mice	Spleen (CIA mice)	FACS	Bulk MDSCs (CD11b^+^Gr-1^+^)	IP ACT (5 × 10^6^), twice weekly Day 35. Established arthritis.	IL-1β → Th17 promotion	Adoptive transfer restored disease after depletion	[19]
CIA	C57BL/6 mice	Spleen (CIA mice)	FACS	M-MDSCs (Ly6C^+^Ly6G^−^)	IV ACT (dose not specified) Days 14, 21. Pre-arthritis.	IL-1β, TNF-α → Th17 promotion	ACT facilitated disease progression; depletion ameliorated disease	[44]
CIA	DBA/1 mice	Spleen (CIA mice)	FACS	PMN-MDSCs (CD11b^+^Ly6G^+^Ly6C^low^)	IV ACT (5 × 10^6^) Days 21, 28. Early-arthritis.	BAFF → TNF-α^+^ B cells via BTK/NF-κB	Adoptive transfer facilitated disease; depletion alleviated severity	[50]
SKG arthritis	SKG mice	Joints (SKG mice)	FACS	CD11b^+^Gr-1^+^	IA ACT (2.5 × 10^4^). Established Arthritis.	Non-canonical NF-κB → osteoclast differentiation	Intra-articular injection promoted synovial inflammation and bone destruction	[38]
CIA	DBA/1 mice	Blood, Spleen, BM (CIA mice)	MACS	Bulk MDSCs (CD11b^+^Gr-1^+^)	Recombinant DKK-1 and neutralizing abs Days 19, 21, 23, 25, 27. Pre/early-arthritis.	DKK-1 → Wnt/β-catenin suppression → MDSC expansion and osteoclast differentiation	DKK-1 promoted MDSC-dependent osteoclastogenesis	[64]
CIA	Human; C57BL/6 mice	BM (CIA mice)	FACS	Mo: PMN-MDSCs (CD11b^+^Ly6C^−^Ly6G^+^), M-MDSCs (CD11b^+^Ly6C^+^Ly6G^−^); Hu: M-MDSCs (CD14^+^HLA-DR^−^/low)	Intramedullary ACT (5 × 10^5^) Days 14, 21. Early-arthritis.	Osteoclast differentiation; RANKL–RANK signaling with Th17	5-FU depletion reduced bone erosion; M-MDSC transfer increased osteoclasts and bone errosions	[66]
CIA	C57BL/6 mice	Spleen, BM (CIA mice)	MACS	Bulk MDSCs (CD11b^+^Gr-1^+^)	IV ACT (5 × 10^6^) Day 14. Pre-arthritis.	IL-6/JAK1/STAT3 → Arg-1 → ↑ IL-17	Sinomenine suppressed Arg-1 and reduced arthritis severity	[49]
RA/AIA	Human; C57BL/6 mice	PBMCs/iMDSCs; splenocytes	FC analysis	Hu: HLA-DR^−^CD33^+^CD11b^+^; Mo: PMN-MDSCs, M-MDSCs	PO triptolide (0.1 µg/g/day) Day 16 post arthritis. Established arthritis.	Arg-1 → ↑ IL-17	Triptolide inhibited MDSC-driven Th17 responses	[54]

Symbols: ↑ increased; → promoted, induced, or resulted in.

## 5. Potential Avenues for Therapeutic Targeting of MDSCs in Inflammatory Arthritis

The paradoxical effects of MDSCs in inflammatory arthritis necessitate therapeutic strategies that account for disease stage, tissue microenvironment, and cellular subset composition. Three broad approaches have emerged from preclinical studies: (1) adoptive transfer of ex vivo-generated suppressive myeloid cells or exosomes, (2) pharmacological reprogramming of pathogenic myeloid populations toward immunosuppressive phenotypes, and/or (3) selective depletion of pathogenic subsets while preserving protective populations. While many of these approaches have shown impressive results in animal models, the historically poor rate of bench-to-bedside translation in autoimmunity warrants cautious interpretation.

### 5.1. Adoptive Cell- and Exosome-Based Therapies

Adoptive cell therapy using ex vivo-expanded suppressive myeloid cells has demonstrated efficacy in preclinical arthritis models. However, further clarification and verification of optimal culture conditions will be critical to maintaining the suppressive mechanistic requirement of IL-10 production [42,45]. Alternatively, MDSC-derived exosomes may provide a cell-free alternative for the delivery of immunomodulatory cargo (miR-29a-3p, miR-93-5p, and PGE2) without the complications associated with in vivo cellular transfusions [69,70]. However, the pharmacokinetics, pharmacodynamics and manufacturing practices of exosomal therapies remain in their nascent stages and therefore should be interpreted with caution. Beyond direct MDSC cellular or exosome approaches, mesenchymal stem cell (MSC) therapy was shown to expand MDSCs and decrease inflammatory cytokines in AIA, although the evidence remains correlative [76].

### 5.2. Pharmacological Reprogramming of MDSCs

Pharmacological reprogramming or promotion of MDSCs offers a more clinically feasible approach when compared to cellular therapies. Indeed, some already approved RA medications have been shown to promote the suppressive anti-inflammatory MDSC phenotype. Proof of concept was first demonstrated with tofacitinib treatment that promoted expansion of MDSCs in the bone marrow and ameliorated arthritis in SKG mice with adoptive transfer [63]. In a separate study using a GPI-induced arthritis model, combination treatment with fingolimod (FTY720) and pathogenic antigen established antigen-specific immune tolerance, attributed in part to the expansion of a highly suppressive CD369^+^CD11b^+^Gr-1^+^ MDSC population [75]. In SKG mice, rapamycin was demonstrated to reprogram 6-diazo-5-oxo-L-norleucine (glutamine metabolism inhibitor) toward a suppressive phenotype capable of inhibiting CD4^+^ T-cell proliferation, and the combination additively ameliorated arthritis with reduced Th17 responses [77].

Other pharmacological approaches have focused on altering specific MDSC signaling pathways to enhance their immunosuppressive capacity. For instance, 3α-aminocholestane (3AC) inhibition of Src homology 2 domain-containing inositol polyphosphate 5-phosphatase 1 (SHIP1) via promoted unopposed PI3K/AKT signaling expanded MDSCs in vivo and attenuated CIA. Adoptive transfer experiments confirmed that 3AC-induced MDSCs (but not naïve MDSCs) were the functional mediators of disease amelioration [72]. The S100A8/A9 alarmin system represents another promising reprogramming target. Exogenous S100A8 treatment induces suppressive myeloid cells in vivo through TLR4-dependent mechanisms and suppresses the inflammatory phenotype in S100A9-knockout mice with severe arthritis [42]. Deoxyinosine, a purine metabolite, was shown to be significantly reduced in RA patient serum and attenuated CIA by expanding immunosuppressive MDSCs and suppressing T-cell proliferation [78].

Taken together, these approaches demonstrate that not all MDSC populations are inherently therapeutic. Moreover, these populations are expandable, and pharmacological priming may be a prerequisite for the ameliorative functions in RA.

### 5.3. Targeting Pathogenic MDSC Pathways

Recent studies demonstrate that small molecules can redirect pathogenic myeloid populations toward protective phenotypes by disrupting the Arg-1/Th17 axis. The alkaloid sinomenine was shown to bind JAK1 to inhibit IL-6-induced STAT3 activation, thereby suppressing Arg-1 expression and blocking myeloid cell-driven Th17 polarization while simultaneously inhibiting receptor activator of nuclear factor κB ligand (RANKL)-induced osteoclastogenesis [49]. Similarly, triptolide reduced Arg-1 expression in Ly6C^hi^ cells and attenuated their capacity to promote Th17 differentiation [54]. Additionally, α-difluoromethylornithine (DFMO), an ornithine decarboxylase inhibitor that blocks polyamine biosynthesis upstream of Arg-1, suppressed CIA by impairing pathogenic MDSC function and reducing Th17 responses [79].

Targeting of pathogenic MDSCs has shown promise in other autoimmune diseases. In SLE, Li et al. demonstrated that Notch1 signaling is aberrantly activated in granulocyte–monocyte progenitors and that the Notch1 inhibitor DAPT reduced MDSC frequencies, decreased ROS production, and attenuated disease progression [61].

### 5.4. Translational Challenges

As briefly mentioned above, a major limitation to existing pharmacological studies is the use of animal models which are known to have fundamentally different MDSC biology. Another question raised by temporality of MDSC intervention in animal models is whether MDSC-based strategies would be effective in patients with established RA, who represent the vast majority of the clinical population. Lastly, several interesting pharmacological treatments with demonstrated effects on MDSCs carry significant side effects and narrow therapeutic windows.

## 6. Perspectives and Emerging Concepts

**Figure 2:**
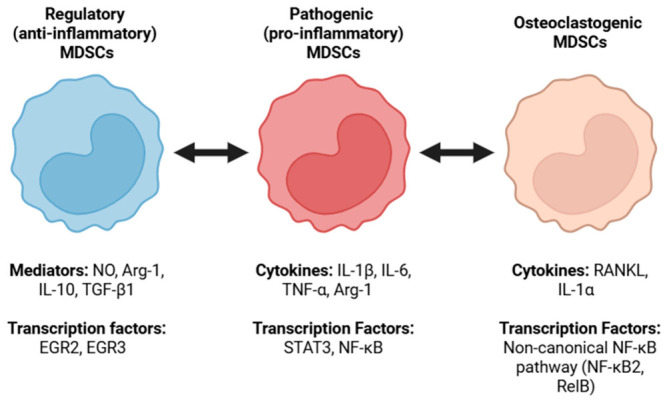
Plasticity and functional polarization of myeloid-derived suppressor cells (MDSCs) during inflammatory arthritis. MDSCs exhibit functional plasticity and can adopt distinct phenotypic states depending on microenvironmental cues. Regulatory (anti-inflammatory) MDSCs produce suppressive mediators, including nitric oxide (NO), arginase-1 (Arg-1), interleukin-10 (IL-10), and transforming growth factor-β1 (TGF-β1), and are associated with activation of EGR2 and EGR3 transcription factors. In contrast, pathogenic (pro-inflammatory) MDSCs secrete cytokines such as IL-1β, IL-6, tumor necrosis factor-α (TNF-α), and Arg-1, and are regulated by signal transducer and activator of transcription 3 (STAT3) and nuclear factor-κB (NF-κB). A third subset, osteoclastogenic MDSCs, is characterized by the production of receptor activator of nuclear factor κB ligand (RANKL) and IL-1α and is driven by the non-canonical NF-κB pathway (NF-κB2, RelB). Schematic created with BioRender.com.

A central observation from studies of MDSCs in inflammatory arthritis is their diametrically opposed functions, with evidence supporting both disease-ameliorating and disease-promoting roles. Taken together, this apparent dichotomy may reflect the existence of functionally distinct MDSC states, rather than experimental inconsistency. This paradigm is somewhat reminiscent of the classical polarization framework described for macrophages, in which pro-inflammatory M1 and anti-inflammatory M2 subsets arise in response to environmental cues. However, the M1/M2 model is now recognized as an oversimplified representation of broader macrophage activation states [80,81]. As shown in Figure 2, one possibility is that MDSCs in inflammatory arthritis exist along a continuum of polarized myeloid states, shaped by the local microenvironment, cytokine milieu, and underlying transcriptional and metabolic programs. If confirmed, this model may reconcile seemingly contradictory findings and establish a coherent foundation for understanding MDSC biology in autoimmunity.

### 6.1. Environmental Cues Drive MDSC Polarization Through Transcriptional and Metabolic Reprogramming

In the context of inflammatory arthritis, the local microenvironment of specific tissues has been implicated in whether MDSCs adopt a regulatory or pathogenic program [38,71]. Tissue-specific cytokines, alarmins and cellular interactions act as instructive cues that shape MDSC fate and effector function within specific disease contexts. For example, duration of the alarmin S100A8/A9, which is highly expressed within the synovium, serves as a critical instructive cue, with prolonged exposure promoting regulatory MDSCs via a TLR4-dependent mechanism to limit arthritis severity [42]. Conversely, joint-resident MDSCs were shown to upregulate the non-canonical NF-κB pathway (*Nfkb2* and *Relb*), which induced a stable functional program for differentiation into bone-resorbing osteoclasts [38]. Cellular interactions via intra-articular B cells were demonstrated to actively prime MDSCs for enhanced osteoclast differentiation, with switched memory B cells exerting the most pronounced effect. In the same study, MDSCs from B-cell-deficient mice showed both reduced osteoclastogenic capacity and altered gene expression profiles [65]. Together, these findings reveal the complexity of the tissue microenvironment to determine MDSC functional fate along a regulatory-to-osteoclastogenic spectrum.

Cross-disease comparisons further illuminate how environmental cues shape MDSC fate. In EAE, lung-resident MDSCs adopt a Th17-promoting phenotype distinct from their splenic counterparts, demonstrating that tissue-specific microenvironments can override the default suppressive program [52]. In SLE, MDSC-derived S100A8/A9 promotes TLR7 pathway activation in macrophages and dendritic cells via an IFN-γ-dependent autocrine loop, converting an alarmin that is protective in arthritis (via TLR4) into a pathogenic signal in lupus (via TLR7) [60].

Collectively, these studies underscore that MDSCs’ functional fate is not intrinsically fixed but is instead dictated by the tissue microenvironment they encounter. They also demonstrate that the same signals can promote pathogenic or protective effects depending on the disease context and local microenvironment. Perhaps the best example is the alarmin S100A8/A9, which drives regulatory MDSC expansion through TLR4-dependent mechanisms to limit arthritis severity, yet promotes pathogenic TLR7 activation in macrophages and dendritic cells via an IFN-γ-dependent autocrine loop in SLE. Thus, disease outcome is not determined by MDSCs alone, but by the context in which they function.

### 6.2. MDSCs Exist as Plastic and Reversible Functional States Rather than Fixed Lineages

Accumulating evidence suggests that MDSCs exhibit substantial functional plasticity, with the capacity to transition between regulatory and pathogenic states in response to changing environmental conditions. As discussed in Section 4.2 and Section 4.3, this plasticity presents a therapeutic opportunity, as pharmacological agents can selectively reinforce suppressive programs or disrupt pathogenic reprogramming without eliminating MDSCs entirely. Thus, rather than representing terminally differentiated lineages, these states likely reflect dynamic and reversible functional changes. This plasticity offers a window for intervention, using immunomodulatory drugs to polarize cells. This model implies that individual MDSCs may shift along a functional continuum over time, challenging static classification schemes and emphasizing the need for longitudinal and fate-mapping approaches to capture in vivo dynamics.

### 6.3. High-Dimensional Immunophenotyping Is Required to Define Distinct MDSC Subsets

Current limitations in defining MDSC populations stem from insufficient phenotypic resolution and overlap with other myeloid cell types. While CD369 (Dectin-1) has been identified as a potential candidate marker for ameliorative MDSCs in inflammatory arthritis [75], single-marker approaches are unlikely to capture the full functional heterogeneity of MDSCs. For instance, integration of scRNA-seq and mass cytometry from RA synovial tissue has defined disease-expanded myeloid states invisible to conventional flow cytometry [40,82].

In cancer biology, scRNA-seq has revealed that MDSCs emerge through aberrant maturation trajectories rather than as discrete lineages, with markers such as CD84 and LOX-1 distinguishing immunosuppressive MDSCs from classical neutrophils [34,35]. The emerging consensus that myeloid cell biology is best understood through functional states governed by metabolic reprogramming rather than fixed lineage identity directly parallels the regulatory-to-pathogenic spectrum observed in inflammatory arthritis MDSCs [83].

Systematic application of these high-dimensional approaches to MDSC populations, combined with functional validation against T helper, Treg, and osteoclastogenic readouts, will be essential to map the full spectrum of MDSC functional states that govern disease outcomes.

## 7. Conclusions

Taken together, the accumulating body of evidence suggests that the paradoxical roles of MDSCs in inflammatory arthritis are best understood not as conflicting observations, but as manifestations of a highly plastic and context-dependent myeloid continuum. This conceptual framework underscores the need to move beyond static phenotypic definitions toward integrative approaches that incorporate transcriptional, metabolic, and functional profiling to resolve MDSC heterogeneity. In the context of inflammatory arthritis and other autoimmune diseases, therapeutic strategies should aim to precisely modulate their functional polarization and enhance regulatory programs while limiting pathogenic potential. Achieving this will require improved tools to define subset-specific biology, identify stable markers of suppressive versus inflammatory states, and map temporal and tissue-specific dynamics in vivo. Ultimately, a deeper mechanistic understanding of MDSC plasticity may enable the rational design of targeted therapies that harness these cells to restore immune homeostasis in inflammatory arthritis and other autoimmune diseases.

## Data Availability

No new data were created or analyzed in this study. Data sharing is not applicable to this article.

## Abbreviations

The following abbreviations are used in this manuscript:

5-FU	5-fluorouracil
ACT	Adoptive cell transfer
AIA	Antigen-induced arthritis
Arg-1	Arginase-1
BAFF	B-cell-activating factor
BM	Bone marrow
CIA	Collagen-induced arthritis
DCs	Dendritic cells
FACs	Fluorescence-activated cell sorting
G-CSF	Granulocyte colony-stimulating factor
GM-CSF	Granulocyte–macrophage colony-stimulating factor
Gr-1	Granulocyte marker 1 (Ly6G/Ly6C)
IA	Intra-articular
IL-1α	Interleukin-1 alpha
IL-1β	Interleukin-1 beta
IL-6	Interleukin-6
iNOS	Inducible nitric oxide synthase
M-MDSCs	Monocytic myeloid-derived suppressor cells
MACS	Magnetic-activated cell sorting
MB	Myeloblasts
MDPCs	Monocyte-dendritic progenitor cells
miR	microRNA
NF-κβ	Nuclear factor kappa B
NO	Nitric oxide
OCs	Osteoclasts
PD-L1	Programmed death ligand 1
PGE2	Prostaglandin E2
PGIA	Proteoglycan-induced arthritis
PMN-MDSCs	Polymorphonuclear myeloid-derived suppressor cells
RANKL	Receptor activator of nuclear factor κB ligand
RNS	Reactive nitrogen species
ROS	Reactive oxygen species
S100A8/A9	S100 calcium-binding proteins A8 and A9
TGF-β	Transforming growth factor beta
Th1	T helper 1 cells
Th17	T helper 17 cells
TNF-α	Tumor necrosis factor alpha
Treg	Regulatory T cells
